# *De novo* assembly and characterization of root transcriptome in two distinct morphotypes of vetiver, *Chrysopogon zizaniodes* (L.) Roberty

**DOI:** 10.1038/srep18630

**Published:** 2015-12-18

**Authors:** Debasis Chakrabarty, Puneet Singh Chauhan, Abhishek Singh Chauhan, Yuvraj Indoliya, Umesh Chandra Lavania, Chandra Shekhar Nautiyal

**Affiliations:** 1 CSIR-National Botanical Research Institute, Rana Pratap Marg, Lucknow 226 001, India; 2 Department of Botany, Lucknow University, Lucknow 226 007, India.

## Abstract

Vetiver, a perennial C_4_ grass, has long been known for its multifarious uses in perfumery, medicine and environmental protection. Two distinct vetiver morphotypes have been identified in India, i.e., A. North Indian type characterized by thick and smooth fast growing roots that produce superior quality of laevorotatory oil; and B. South Indian type with more number of thin and hairy roots that produce inferior quality of dextrorotatory oil. The two morphotypes were targeted for transcriptome analysis to understand the contribution of genetic background on oil quality and root morphology. Sample A showed enhanced activity of flavonoid and terpenoid biosynthesis related genes, i.e. ERF, MYB, bHLH, bZIP and WRKY. Interestingly, expression analysis revealed that the genes involved in sesquiterpene biosynthesis pathway were up regulated in Sample A. Moreover, some of the genes involved in mevalonate pathway of sesquiterpene biosynthesis were unique to Sample A. Our results also demonstrated several transcripts involved in root development and hormonal regulation being up regulated in Sample A. To validate gene expression results of RNA-seq data, 20 transcripts were validated by qRT-PCR experiment. The present study provided an important start point for further discovery of genes related to root oil quality in different ecotypes of vetiver.

Vetiver (*Chrysopogon zizaniodes* (L.) Roberty; syn.: *Vetveria zizaniodes* (L.) Nash.) is a perennial densely tufted C_4_ grass native to India. The name “vetiver” is derived from the Tamil word “vettiver”. It is called “vetivert” in Reunion Island where it is naturalized and is said to have been introduced from India through Indonesia. Vetiver is a miracle grass^1^ that can tolerate wide range of climatic and soil conditions, ranging from dry to submerged conditions, 4–50 °C atmospheric temperature and pH range of 4–11. This grass, traditionally used for extraction of essential oil from its roots, has attracted global attention as a natural and inexpensive resource for multifarious environmental applications, including conservation and detoxification of degraded soil and water and also for mitigation of flood and landslide disasters^2^. The grass is now grown all across the globe from tropical to Mediterranean climate. Lately, Lavania and Lavania^3^ proposed a “vetiver grass model” for sequestration of atmospheric carbon into subsoil horizons to mitigate global warming. The vetiver grass bears prolific clump of tillers, long leaves, an erect paniculate inflorescence and deep penetrating tufted fibrous roots. Apart from the traditional use of its essential oil in perfumery and medicine, the hedges of vetiver have been used for contour protection in India since centuries^2^. Indians were the first to recognize its aromatic and medicinal uses, followed by its other cottage and environmental uses in India and elsewhere^2^,^4^. In India, it is found in its natural state throughout the tropical and subtropical plains and lower hills, particularly along the river banks and marshy lands where it grows like a weed. It has wide range of ecological distribution ranging from sandy sea coasts and swamps to plains and foothills, and also on the hilltops up to elevations of 800 m in the Kumaun hills of Uttarakhand in India.

Two distinct morphological complexes of vetiver are found to inhabit spatially separated geographic regions in India: one in the north along the Indo-Gangetic plains and adjoining areas mainly in the states of Rajasthan, Madhya Pradesh, Uttar Pradesh, Bihar and West Bengal, and the other in the south along the east and west coasts of Indian peninsula in the states of Andhra Pradesh, Karnataka, Tamil Nadu and Kerala^4^. The two forms are karyomorphologically distinct and reproductively differentiated geographic complexes^5^. The north Indian wild types are profusely flowering, high seed-setting and produce superior quality of laevorotatory root oil (ruh-khus or khus oil) whereas the south Indian cultivated types are low/late flowering, low/non seed-setting and produce inferior quality of dextrorotatory root oil (vetiver oil)^6^. Extensive work on evaluation of genetic diversity, genetic analysis, genetic improvement has been done on Indian vetiver, and a good number of superior clones, north × south hybrids and artificial polyploids have been segregated for high productivity of essential oil and high value perfumery notes ranging from earthy-to-rosy-to- saffron odour^4^,^7^.

During the last few years, a large number of transcriptomic and genomic sequences became available in several model organisms, which have greatly improved the understanding of the complexity of physiological processes in higher plants^8^,^9^,^10^,^11^. Despite the commercial and medicinal importance of vetiver, little genomic research has been done on this species. For vetiver, only a small set of of 32 EST sequences are available in GenBank database (http://www.ncbi.nlm.nih.gov/genbank/). This small public domain data are not sufficient for elucidating the molecular mechanisms controlling the traits of interest. Therefore, extensive genomic and transcriptomic sequence data are needed for vetiver, which can be used for discovering new genes related to root structure and oil quality in different ecotypes and for developing high density microarrays for further characterization of gene expression profiles.

In the present study, we have carried out transcriptome sequence analyses in two strategically selected and contrasting morphotypes of vetiver, one representing the North Indian type having thick, smooth and fast growing roots, and the other the South Indian type having thin, hairy and more roots. We have applied Illumina paired-end sequencing technology to characterize the root transcriptome of vetiver and to develop SSR markers. *De novo* assembly for both samples was carried out using Trinity. Functional categorization and pathway analysis were also performed. This is the first report on a global comparative molecular analysis of root transcriptome in two distinct morphotypes of vetiver using Illumina paired-end sequencing. This dataset will serve as a public information platform for gene expression, genomics, and functional genomics studies in vetiver.

## Results and Discussion

### Morphometric differentiation of the two contrasting morphotypes

**Figure 1:**
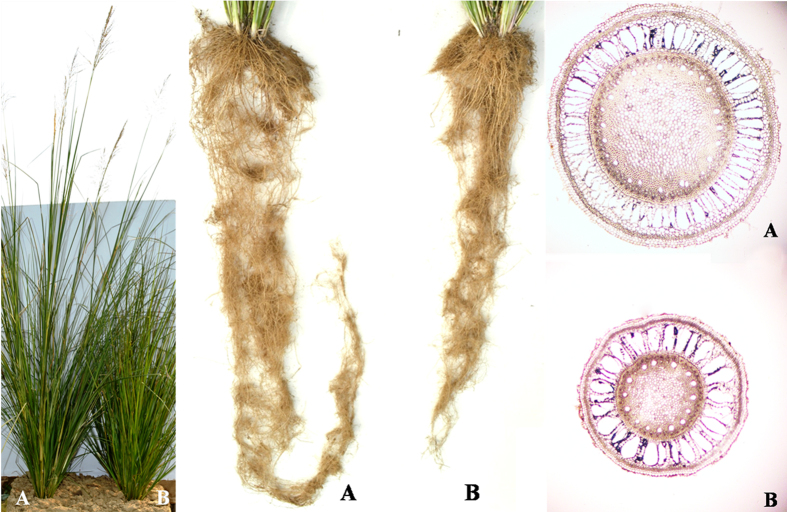
Morphology of whole plants, root architecture and anatomy of two vetiver morphotypes, South Indian Type (A) and North Indian Type (B).

Vetiver is a tall (1–2 m), fast-growing, perennial tussock grass^12^,^13^. It has a long, massive tufted root system, which can penetrate the deeper layers of the soil. Its roots produce an essential oil that is considered as a perfume in its own right, and is highly valued in the perfume industry. Vetiver oil is rich in sesquiterpenes, and is psychologically grounding, calming, and stabilizing. In India there are two distinct Vetiver morphotypes, North Indian Type and South Indian Type. North Indian types are of profuse flowering, high seed forming with thicker roots (root diameter is around 2 cm) while leaves are broader (lamina width at the leaf base around 2 cm). It has lesser number of roots per tiller compared to the South Indian type. North Indian type has high quality essential oil enriched with ketones and aldehydes that add to its perfumery value. North Indian type vetiver plants are tall reaching up to 1.6 m, with inflorescence stalk reaching up to 2.5 m. South Indian type sports higher number but thin roots, and thin leaves while root diameter is around 1.2 mm, lamina width near leaf base is 1–1.2 cm, plant height up to 1.2 m and inflorescence stalk reaching 1.5 m. South Indian morphotypes are low or late flowering and low seed forming. Roots are rich in essential oil concentration but the oil is of poor quality and having positive optical rotation. In order to ensure best morphological distinction in the two morphotypes we identified two distinct clones i.e. Sample A and Sample B, representing North and South Indian geographical complexes, respectively for the present study. A brief account of their morphotypic differentiation depicting key features is provided in Table 1, Fig. 1.

### Sequencing of vetiver root transcriptome

To generate a broad survey of genes associated with root morphology and difference of oil quality of two different morphotypes, North Indian and South Indian type, a total of 6,33,37,598 raw sequencing reads with 101 bp were generated from a 200 bp insert library. An assembler, Trinity was employed for *de novo* assembly. After stringent quality check and data cleaning, approximately 5,67,50,434 million high quality reads were obtained with 89.60% Q20 bases (base quality more than 20). Based on the high quality reads, a total of 73,061 contigs were assembled with an average length of 101 bp. The length of contigs ranged from 201 to 12,238 bp. The NGSQCTOOLKIT^14^ results are given in Table 2A,B.

### *De novo* assembly and functional annotation

**Figure 2:**
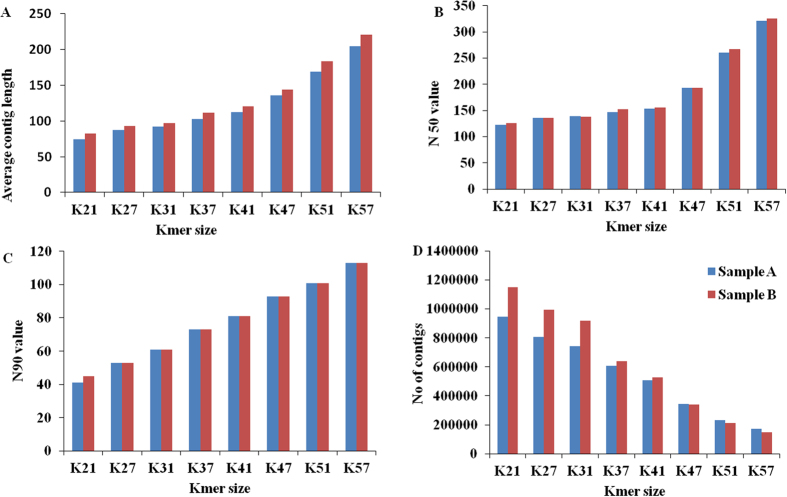
Comparison of *de novo* assembly of two samples using velvet program as a function of K mer length. (**A**) Average contig length, (**B**) N50 value, (**C**) N90 value, (**D**) Number of contigs. Blue color indicates Sample A while Red color indicates Sample B.

The *de novo* assembly of vetiver transcriptome was optimized after assessing the effect of various assembly parameters, trimming bases at sequence read ends and different assembly programs as described in materials and methods. We got ~70% high quality sequence reads that were assembled using Velvet program^15^ at different k-mer length of 21, 27, 31, 37, 41, 47, 51 and 57 (Fig. 2A–D). We analysed various output parameters like average contig length, N50 value, N90 value and number of contigs as a function of k-mer length. The results suggested that k-mer length inversely affects the number of contigs. We found the best assembly to be that for k 57.

**Figure 3:**
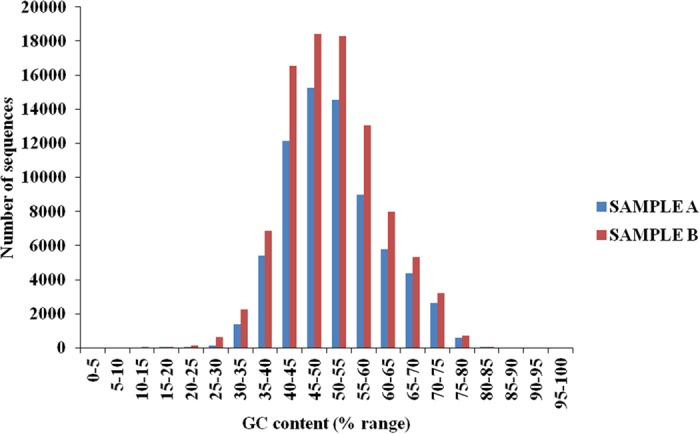
GC content analysis of vetiver transcripts showing abundant number of sequences in sample B compare to Sample A. GC content was calculated and percentage of transcripts with GC content within a range are represented.

**Figure 4:**
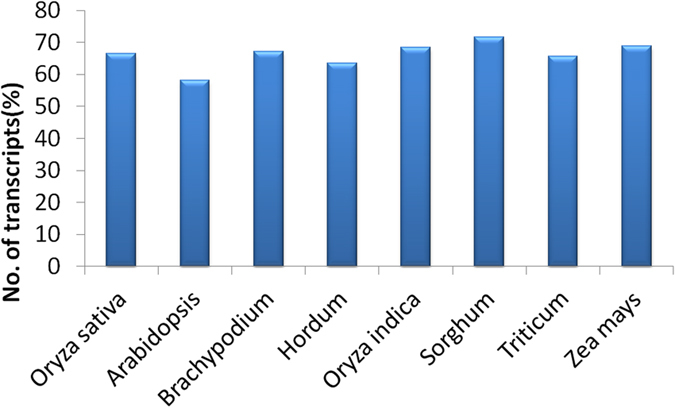
Sequence conservation of vetiver transcripts with annotated proteins of completely sequenced other plant species showing maximum similarity with *Sorghum*. The percentage of transcripts showing significant similarity (E- value ≤ 1 E −05) in BLASTX.

Vetiver transcriptome sequencing data was analysed in terms of GC ratio as the latter plays a vital role in gene and genome regulation and also in determining the physical properties of the genome and nucleic acid stability as well^16^,^17^. As Fig. 3 indicates, number of sequences and GC content was more between 45–50 and 50–55 in both the samples. We analyzed the sequence conservation in vetiver transcripts with proteome sequences of Poaceae members like *Oryza sativa* subsp. indica, *O. sativa* subsp. japonica, *Sorghum bicolor*, *Hordeum vulgare*, *Brachypodium distachyon, Triticum aestivum*, *Zea mays*, and that of *Arabidopsis thaliana* as a dicot representative. Among all, *Sorghum* shows highest similarity (71.60%) with vetiver transcript followed by *Zea mays*, *Bracypodium* and *Oryza*. *Arabidopsis thaliana*, being a dicot, showed least similarity (58.11%) with vetiver transcript (Fig. 4). The sequence comparison result supports previous report of Adams *et al.*^18^ about *Sorghum*, as the most closely related genus of *Chrysopogon.*

### Differential gene analysis

**Figure 5:**
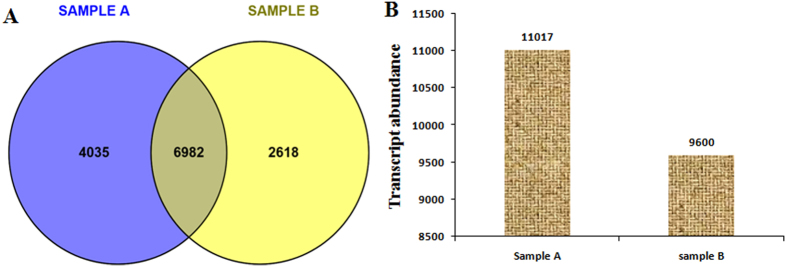
Analysis of global gene expression among two samples. (**A**) Venn diagram showing 6,982 common transcripts detected among sample A and sample B. (**B**) Number of genes expressed in each of the samples (11,017 transcripts for sample A and 9,600 transcripts for sample B).

**Figure 6:**
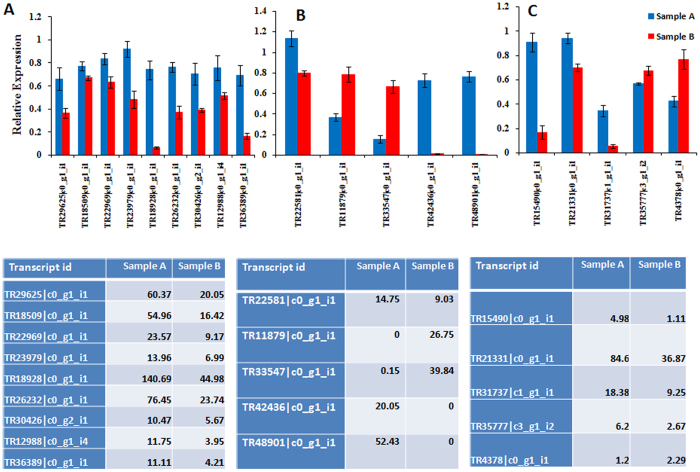
Validation of transcriptomic data of selected genes with qRT PCR among Sample A and Sample B of Vetiver. Relative expression for all genes, in all samples were calculated using 2^-delta Ct. (**A**) Terpenoid biosynthesis related transcripts, (**B**) Differential expressed transcripts, (**C**) Root specific transcripts. Tables showing transcriptomic expression value (FPKM) of different validated groups (**A–C** respectively). Bars show the mean of triplicate samples and error bars represent the SD.

Differential gene analysis is one of the important data analysis strategies for expression studies of the transcripts. It also involves various approaches to determine whether counts for a transcript or exons are significantly different across experimental conditions. In order to analyse differentially expressed genes, edgeR program was used as described in materials and methods^19^. We got 65,779 differential genes from the edgeR program (Table S3). In total, we detected 13,635 significant transcripts expressed in both samples, on the basis of FDR (q value < 0.05). The distribution of these genes is presented in a Venn diagram (Fig. 5A), which shows that 6,982 genes were common among both samples. The number of unique genes detected was higher in Sample A than inSample B. Additionally, several hundred highly expressed transcripts were detected in Sample A as compared with Sample B (Fig. 5B). To experimentally confirm that the transcripts obtained from sequencing data were indeed expressed, 20 transcripts related to terpenoid biosynthesis, root specific and randomly selected genes were chosen for qRT-PCR analysis. A very good correlation was obtained by real-time PCR analysis with respect to transcriptomic data (Fig. 6). These results further confirmed the potential of NGS technologies to quantify gene expression.

### Differential expression analysis of transcripts encoding putative transcription factors

**Figure 7:**
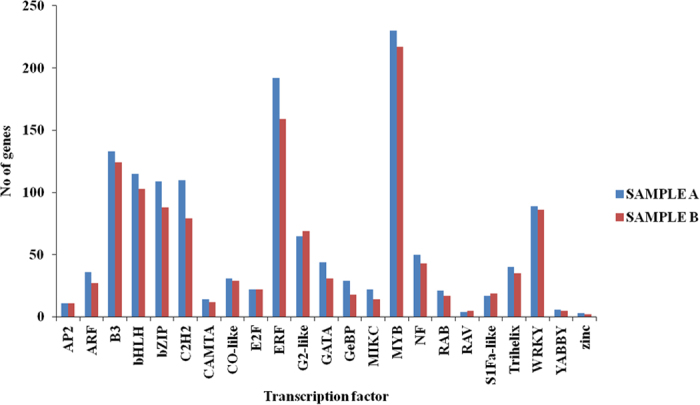
Distribution of vetiver transcripts in different transcription factor families showing over representation in sample A compare to sample B.

Transcription factors modulate gene expression by interacting with the promoter regions of related genes. There are several transcription factors (TFs) known to be involved in regulation of terpenoid biosynthesis pathways. Therefore, analysis of transcriptional factors in contrasting morphotypes was carried out by sequence comparison with known transcription factors gene families. As shown in Fig. 7, several transcription factors like ERF, MYB, B3, bHLH, bZIP and WRKY were overrepresented in Sample A. Associative modulation of several plant processes suggest involvement of transcription factors (TFs) for coordinated regulation of gene expression. Some transcription factors like APETALA2 (AP2) are involved in terpene indole alkaloid biosynthesis^20^, and WRKY1 in sesquiterpene biosynthesis^21^,^22^. Very recently two JA-responsive AP2 family transcription factors (AaERF1 and 2) were found to regulate *Amorpha-4,11-diene synthase* (*ADS*), a sesquiterpene synthase involved in the biosynthesis of artemisinin^23^, and members of the MYB, bZIP and WRKY transcription factor families involved in the regulation of stress responses^24^,^25^,^26^. Higher expression of above mentioned transcription factors in Sample A may be associated with terpene biosynthesis and probably thus related with good quality oil in sample A as compared to sample B.

### Gene ontology and pathway analysis

**Figure 8:**
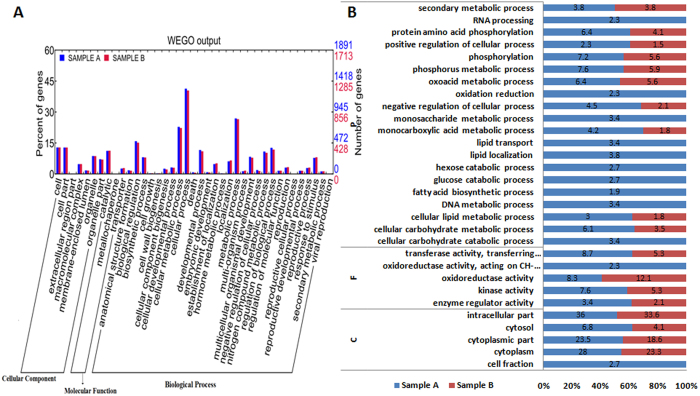
Functional annotation of vetiver transcripts. GOSlim term assignment to the Vetiver transcripts in different categories of biological process (P), molecular function (F) and cellular component (C). (**A**) Wego online tool showing transcript abundance in sample A compare to sample B. (**B**) GO annotation of some selected processes retrieved from AgriGO. Blue color indicates percentage of genes involved in sample A and Red indicate percentage of genes involved in sample B.

Gene ontology (GO) and pathway analysis were performed in order to classify the assembled transcripts. The genes with more than 10 FPKM were considered for GO and pathway analysis. Thus 11,017 genes from Sample A and 9,600 genes from Sample B were used for further analysis. WEGO ontology tool was used for gene ontology^27^. As shown in Fig. 8A, Sample A has more functions in comparison to sample B. In the cellular component, the most enriched classes were cell, cell part and organelle. The highest percentage of molecular function in GO terms was in catalytic activity. In the biological processes, where major subcomponents were taken, the majority of the GO terms were grouped into two categories- those of metabolic and cellular processes. In detailed GO analysis (Fig. 8B, Table S4A,B), lipid metabolism, transport, DNA metabolism, carbohydrate metabolism and catabolism related genes were overrepresented in Sample A. Additionally, biological processes, like secondary metabolism, phosphorylation, oxoacid metabolism, though present in both the samples, were found overrepresented in sample A. Among cellular components, cytosol, intracellular part, cytoplasmic fraction and cytoplasm were overrepresented in sample A as compared to Sample B.

**Figure 9:**
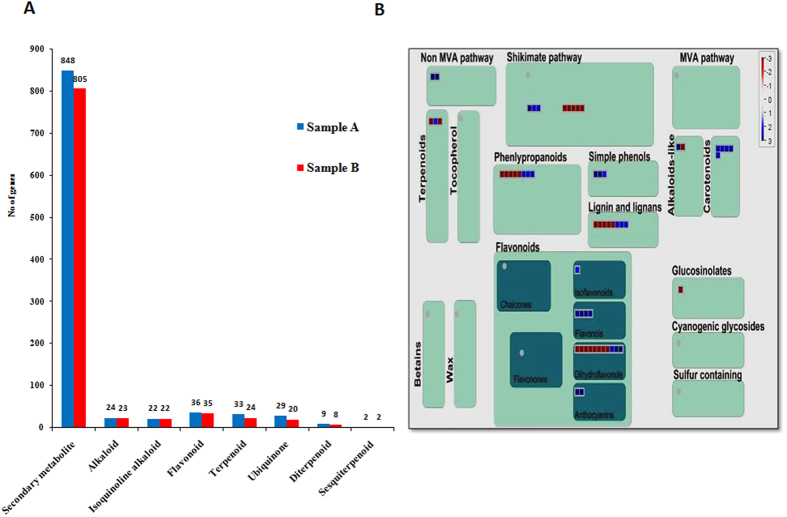
Expression analysis of putative genes involve in secondary metabolism. (**A**) Pathway assignment based on KEGG showing classification based on secondary metabolite categories. (**B**) MapMan visualization showing the observed differential expression patterns of transcripts involve in secondary metabolism, based on the Log2FCs of transcript levels, in Sample B (South Indian Ecotype) compare to Sample A (North Indian Ecotype). In the display, each BIN or sub BIN is represented as a block where each transcript is displayed as a square. Red color indicate down regulation while blue colors showing up regulation in Sample B compare to Sample A.

**Figure 10:**
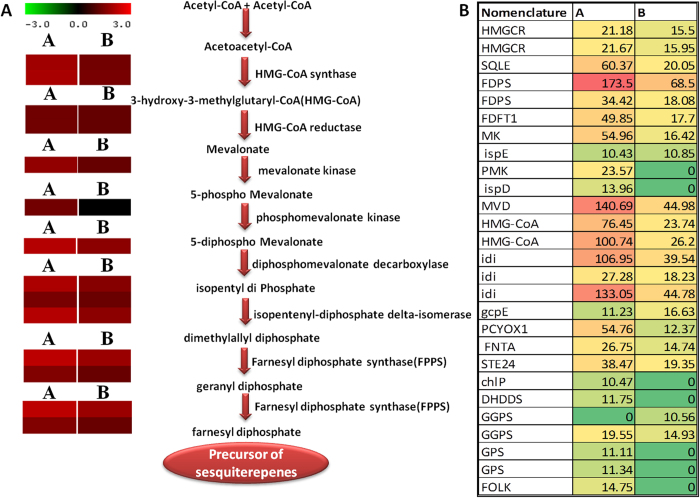
Expression analysis of putative enzymes involved in terpene biosynthesis. (**A**) Putative pathway for sesquiterpene biosynthesis in vetiver. All the enzymes found in this study related to different steps are shown between the reactions catalyzed. Expression of different transcripts related to these enzymes in sample A and Sample B is shown by heatmap. (**B**) Names and expression value (FPKM) of enzymes related to terpene biosynthesis.

Finally, the EC numbers were classified in KEGG pathways^28^, enabling the presentation of enzymatic functions in the context of the metabolic pathways involved in terpenoid biosynthesis. Among the pathways identified, secondary metabolite related transcripts were over- represented (Fig. 9A, Table S5A,B). Moreover, Sample A showed more secondary metabolite - flavonoid, and terpenoid related transcripts compared to sample B. In order to check differential expression pattern of putative genes associated with secondary metabolism, MapMan analysis was performed. Several transcripts were differentially expressed among Sample A and Sample B (Fig. 9B). Genes involved in terpene biosynthesis showed overexpression in Sample A as compared to Sample B, which further confirmed over representation of terpenoid biosynthesis pathway in Sample A. Interestingly, expression analysis of genes involved in sesquiterpene biosynthesis pathway were up-regulated in Sample A as compared to Sample B (Fig. 10A). Interestingly, some of the genes, involved in mevalonate pathway of sesquiterpene biosynthesis, were uniquely present in Sample A while others were over expressed in Sample A as compared to Sample B (Fig. 10B). Further validation of some of the selected genes involved in terpenoid biosynthesis was carried out using qRT-PCR (Fig. 6A, Table S6). In the terpenoid pathway, vetiver homologues of TR29625|c0_g1_i1 (squalene monooxygenase), TR18509|c0_g1_i1 (mevalonate kinase), TR22969|c0_g1_i1 (phosphomevalonate kinase), TR23979|c0_g1_i1 (2-C-methyl-D-erythritol 4-phosphate cytidylyltransferase), TR18928|c0_g1_i1 (diphosphomevalonate decarboxylase), TR26232|c0_g1_i1 (hydroxymethylglutaryl-CoA synthase), TR30426|c0_g2_i1 (geranylgeranyl reductase), TR12988|c0_g1_i4 (ditrans, polycis-polyprenyl diphosphate synthase) and TR36389|c0_g1_i1 (geranyl diphosphate synthase) were expressed much higher in Sample A roots than in Sample B. Plants use both the mevalonate dependent and the mevalonate-independent, or deoxyxylulose 5-phosphate (DXP), pathways for isoprenoid synthesis 29,30,31,32,33. Over representation and higher expression of genes involved in mevalonate dependent sesquiterpene biosynthesis in Sample A, may be responsible for better quantity and quality of vetiver oil in Sample A as compared to Sample B.

### Expression analysis of transcripts invoved in hormonal regulation and root development

**Figure 11:**
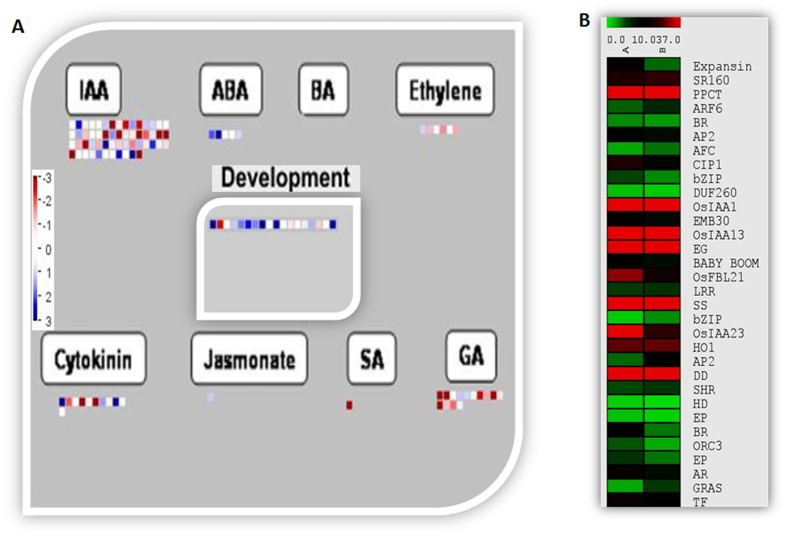
Expression analysis of transcripts involved in regulation and root development. (**A**) MapMan visualization showing the observed differential expression patterns of transcripts involve in hormonal regulation and development, based on the Log2FCs of transcript levels, in Sample B (South Indian Ecotype) compare to Sample A (North Indian Ecotype). In the display, each BIN or sub BIN is represented as a block where each transcript is displayed as a square. Red color indicate down regulation while blue colors showing up regulation in Sample B compare to Sample A. (**B**) Heat map showing FPKM based expression of different transcripts involve in root development and regulation. Red and Green colors showing lower and higher expression of related transcripts, respectively.

As mentioned earlier, among the two morphotypes examined Sample A produced good quality vetiver oil and possessed a better root system architecture than the Sample B. In order to check expression pattern of some of the transcripts involved in root morphology and development, candidate genes analysis was carried out (Table S7). Results suggested that several transcripts involved in root development were differentially regulated among both the morphotypes. Phytohormones play an important role in root development. In order to check expression pattern of various phytohormones responsive genes, MapMan mediated visualization was carried out and as shown in Fig. 11A, several candidate genes involved in auxin and cytokinin mediated regulation were differentially expressed in both morphotypes. Additionally some of the ABA, GA and Ethylene responsive genes were also showed differential expression pattern among both samples. Some of the candidates genes involved in root development were also found to be differentially expressed in the two morphotypes. Based on the literature expression pattern of putative transcripts involved in root development and regulation were also analysed. Several genes, directly or indirectly involved in root architecture and development, were found differentially regulated in Sample A than in its counterpart (Fig. 11B and Table S8). Our data also suggests that several auxin responsive transcripts were also up regulated in Sample A than in Sample B (Table S7). These transcripts have been reported to play an important role in root development. Products of *OsORC3 (*Origin recognition Complex 3) and *OsCYP2* are involved in lateral root initiation and development in rice^34^,^35^ (34–35). Similarly, auxin efflux carrier (*EIR1)*, a root-specific protein involved in auxin transport, is required for root development and gravitropism in *Arabidopsis thaliana*^36^. Some of the selected genes were further validated using qRT-PCR (Fig. 6C). Among the genes tested for q-RT-PCR, the expression levels of TR15490|c0_g1_i1 (Similar to *PLETHORA1*) and TR35777|c3_g1_i2 (auxin transport protein *REH1*) showed very good correlation with the transcriptome data. Overexpression of such transcripts may be responsible for the dense and deep root system of Sample A compared to sample B, which in turn leads to greater oil quality by an unknown link between its morphological superiority and better oil quality. Further study may explore such type of correlation in a better way.

### SSR identification

The transcript based SSR markers play an important role in determining functional genetic variations^37^. SSR markers are polymorphic in nature, easy to develop and are a rich source of diversity. We use MISA perl script to find SSR marker in vetiver. A total of 73,061 sequences (Total size of sequences was 5,80,98,639 bp) were examined for SSR identification (Table 3). Out of those, total number of identified SSRs was 13,527, whereas number of SSR containing sequences were 11,240. Additionally, following the criteria used to identify these SSRs, tri-nucleotide repeats were the most abundant (6,295) and hexa-nucleotides were the least abundant (70).

In this study, we utilized Illumina paired-end sequencing technology to characterize the root transcriptome in two different morphotypes of vetiver and to develop SSR markers. Non-normalized cDNA collections from two different types of roots were used to generate a broad survey of genes associated with different morphological characters as well as secondary metabolites. To the best of our knowledge, this study is the first exploration to characterize the root transcriptome of vetiver through the analysis of large-scale transcript sequences resulting from Illumina paired-end sequencing. North Indian (Sample A) morphotype has good quality of oil as well as root system architecture due to over represented transcripts of secondary metabolite and root specific traits as compared to south Indian morphotype. Terpenoid, particularly sesquiterpene biosynthesis pathway played a major role in quality and quantity of Vetiver oil as evident by our transcriptome study. Functional characterization of some of the important genes will lead to complete understanding of Vetiver oil biosynthesis pathway.

## Methods

### Plant material

Two distinct morphotypes representing north and south Indian vetiver that differ for various biological characteristics (Table 1) were targeted for the study. The two distinct forms were segregated from the large collection of vetiver grown at the CSIR-Central Institute of Medicinal and Aromatic Plants, Lucknow, India after ascertaining the distinctness in growth/reproductive behavior and other biological and botanical features grown over several years. Root sample of both varieties were collected from the 15-day-old seedlings grown in autoclaved mixture (1:1). At least three independent biological replicates of each tissue sample were harvested, all the samples were ground in liquid N_2_ and stored at −80 °C for further analysis.

### RNA isolation and quality controls

Frozen tissues were ground to a fine powder in liquid nitrogen and total RNA was extracted using RNeasy plant Mini Kit (QIAGEN, MD) and treated with RNase free DNaseI (QIAGEN, MD) according to manufacturer’s instructions. The quality and quantity of total RNA were analysed by agarose gel and spectrophotometric analysis (ND1000 Nanodrop, NanoDrop Technologies, USA). The equal amount of total RNA from three different preparations were pooled and used for further processing. Quantity as well as quality of pooled RNA was again checked on Agilent 2100 Bioanalyzer RNA chip (Agilent Technologies Inc., Santa Clara, CA). Only the RNA samples with 260 of 280 ratio from 1.8 to 1.9, 260 of 230 ratio from 2.0 to 2.5 and RIN (RNA integrity number) more than 9.0, were used for the analysis

### Illumina sequencing

The cDNA libraries were generated using mRNA assay for sequencing on IlluminaHiSeq2000 sequencing platform. Paired-end cDNA library was generated from root sample and sequencing was performed to generate the ~101 bp paired-end reads. Many quality controls and adaptor removal were done by NGSQCTOOLKIT (http://www.nipgr.res.in/ngsqctoolkit.html) software. This software was used for filtering of high quality reads based on quality score (Q > 20) value given in fastqfile. Removal of adaptor and trimming of reads were done by NGSQCTOOLKIT.

### *De novo* assembly

The assembly was performed on workstation with 12 processor and 56 GB random access memory. We used Trinity (http://trinityrnaseq.github.io), Velvet (http://www.ebi.ac.uk/~zerbino/velvet/) and SOAPdenovo (http://soap.genomics.org.cn/soapdenovo.html) software for *de novo* assembly^14^,^38^,^39^. Trinity working bench was divided into three steps. First one is Inchworm in which construction of de Bruijn graph takes place. Construction of contigs takes place by k-mers. Second one is Chrysalis in which reads partitioning is done by overlapping contigs of inchworm. Third one is Butterfly which is used for traversal of graph by generated reads. deBruijn graph algorithm was used for assembly of short reads. We got good assembly results with our data set. Many assembly parameters were also optimized for best results.

### Functional annotation and similarity search

To find putative functions, vetiver non redundant transcript data set was subjected to blastx against the non-redundant protein sequence database of PlantGDB (ftp://ftp.plantgdb.org/pub/FASTA_187). Proteome dataset was downloaded from plant ensembl (http://plants.ensembl.org/info/website/ftp/index.html) database. The vetiver transcripts were searched against proteome sequence. The results of best hits from both databases were taken with an E-value ≤ 1E-05, considered as significant (Table S1). To analyze the closeness of vetiver with respect to other plant species, we performed blastx with *Arabidopsis thaliana, Brachypodium distachyon, Hordeum vulgare, Oryza sativa* subsp. japonica*, Oryza sativa* subsp. indica*, Sorghum bicolor, Triticum aestivum* and *Zea mays*. Identification of transcription factor was done by blastx using PlantTFDB (http://planttfdb.cbi.pku.edu.cn).

### Differential gene expression analysis

Differential gene expression analysis was carried out using trinity in build tool, EdgeRbioC in R version (http://www.bioconductor.org/packages/release/bioc/html/edgeR.html). After running trinity assembly steps, abundance estimation was performed using RSEM. Finally EdgeR bioconductor was run for differential gene expression analysis. The data were visualized and figures were produced using the MapMan software. A downloadable Version 3.6.0RC1 is available at http://mapman.gabipd.org/web/guest.

### GC content and SSRs identification

GC content analysis was done between two samples. GC content was analysed using Perl program from sample wise assembled contig files. MISA tool (http://pgrc.ipk-gatersleben.de/misa) was used for SSRs identification. Parameters like mono-nucleotide’s repeats more than ten times, di-nucleotide repeats more than six times, tri, tetra, penta, hexa-nucleotide repeats more than five times were considered as MISA search criteria.

### Gene ontology and pathway analysis

To perform gene ontology and pathway analysis, we used transcripts which have FPKM more than 10. Genes were selected from differential file generated using EdgeRbioC described in section2.6.Gene ontology analysis was performed using agriGO search tool (http://bioinfo.cau.edu.cn/agriGO/analysis.php) with singular enrichment analysis statistical test. Sorghum (*Sorghum bicolor* L.) reference genome was taken for gene ontology studies. These GO terms were used in WEGO tool (http://wego.genomics.org.cn/cgi-bin/wego/index.pl) for plotting GO annotation for molecular function, biological process and cellular component analysis. Pathway analysis performed using KEGG database (http://www.genome.jp/kegg). KO ids for pathway analysis generated with the help of KAAS (www.genome.jp/tools/kaas/) by taking *Oryza sativa* as reference genome^40^.

### Expression analysis using qRT PCR

Real time PCR was performed in 20 μl for a set of selected genes using Fast SYBR Green PCR Master Mix (ABI, USA). The list of selected genes and oligonucleotide primers (Eurofins, India) used for each gene are listed in Table S2. Oligonucleotide primers for vetiver actin gene (Table S2) were used as the internal control for establishing equal amounts of cDNA in all reactions. The reactions were performed using the following cycle conditions, an initial 94 °C for 2 min, followed by 30 cycles of 94 °C for 30 s, 60 °C for 30 s, and 72 °C for 30 s, and the final 5 min extension at 72 °C. After obtaining the ct value for each reaction, the relative expression was calculated by 2^-delta Ct method.

### Data access

The illumina sequencing reads of both samples have been submitted to NCBI BIO PROJECT (PRJNA292937) accession number SRP062463. The BIOSAMPLE accession number for Vetiver Sample A (North Indian ecotype) is SRR2167610 and for Sample B (South Indian ecotype) is SRR2167619.

## Additional Information

**How to cite this article**: Chakrabarty, D. *et al.* *De novo* assembly and characterization of root transcriptome in two distinct morphotypes of vetiver, *Chrysopogon zizaniodes* (L.) Roberty. *Sci. Rep.* **5**, 18630; doi: 10.1038/srep18630 (2015).

## Supplementary Material

Supplementary Table S2

Supplementary Table S1

Supplementary Table S3

Supplementary Table S4A

Supplementary Table S4B

Supplementary Table S5A

Supplementary Table S5B

Supplementary Table S6

Supplementary Table S7

Supplementary Table S8

## Figures and Tables

**Table 1:** Morphometric differentiation of the two contrasting Vetiver morphotypes.

Phenotype	Sample A (North Indian type)	Sample B (South Indian type)
General morphology	Tall, broad leaves, profuse and early flowering, high seed set, smooth roots	Dwarf, narrow leaves, low and late flowering, low seed set, hairy roots with profuse secondary roots
Growth Habit	Fast growing	Slow growing
Plant height (taken as leaf length)	172 cm	105 cm
Inflorescence stalk length (culm and inflorescence combined)	205 cm	185 cm
Culm length	100 cm	108 cm
Leaf color	RHS 138A Green Group	RHS 143A Green group
Leaf texture	Stiff	Stiff
Leaf blade stomatal index	4.47	2.37
Leaf base stomatal index	3.42	3.28
Stomatal conductance	0.698 mol/m^2^/sec	0.382 mol/m^2^/sec
Inflorescence : Peduncle length/panicle	65/30 cm	58/25 cm
Seed set and germination	50%	8–10%
Oil Content (in fresh roots)	0.7%	0.4%
Oil quality	Rich in Sesquiterpine alcohols other than Khusimol and Khusinol (50%) with little ketones	Lacks Sesquiterpine alcohols but enriched with 8–10% Ketones (including α-/β-vetivone and nootkatone)
Average shoot/root Length (cm) after 5 months	153/185 cm	105/115 cm
Average root diameter (at the base of main root)	0.249 cm	0.146 cm
Total root dry weight (g/plant) after 5 months	43 g	29 g
Number of slip tillers/primary roots after 5 months	17/208	23/289
Shoot yield (culm/leaf dry matter) after 5 months	99 g	53 g
Carbon content Roots	43% dry wt.	44% dry wt.
Carbon content Shoots	37% dry wt.	38% dry wt.
Photosynthesis efficiency i.e. CO_2_ exchange rate at 10–15 μmol/m^2^/sec photosynthetic active radiation	7.634 μmol/m^2^/sec	4.2 μmol/m^2^/sec
Chlorophyll content	2.386 μg/g fresh wt.	2.701 μg/g fresh wt.
Cytological differentiation	2n = 20	2n = 20
Chromosome morphology (Range in Chromosome size)	2.1 to 4.4 μm	1.9 to 3.7 μm
Haploid chromatin length	29 μm	27 μm

**Table 2:** A NGSQCTOOL kit result for Sample A. Table B NGSQCTOOL kit result for Sample B.

File name	Sample_A_R1.fastq	Sample_A_R2.fastq	Sample_A_R1.fastq_filtered	Sample_A_R2.fastq_filtered
Minimum read length	101	101	101	101
Maximum read length	101	101	101	101
Average read length	101	101	101	101
Total number of reads	3,16,68,799	3,16,68,799	2,83,74,854	2,83,74,854
Total number of reads with non-ATGC bases	36,995	1,26,987	14,586	59,585
Percentage of reads with non-ATGC bases	0.12%	0.40%	0.05%	0.21%
Total number of bases	3,19,85,48,699	3,19,85,48,699	2,86,58,60,254	2,86,58,60,254
Total number of HQ bases	3,06,00,95,276	2,97,07,81,787	2,82,34,69,395	2,81,95,97,570
Percentage of HQ bases	95.67%	92.88%	98.52%	98.39%
Total number of non-ATGC bases	40,345	35,35,607	15,152	1,32,394
Percentage of non-ATGC bases	0.00%	0.11%	0.00%	0.00%
**File name**	**Sample_B_R1.fastq**	**Sample_B_R2.fastq**	**Sample_B_R1.fastq_filtered**	**Sample_B_R2.fastq_filtered**
Minimum read length	101	101	101	101
Maximum read length	101	101	101	101
Average read length	101	101	101	101
Total number of reads	3,31,86,436	3,31,86,436	2,91,16,148	2,91,16,148
Total number of reads with non-ATGC bases	48,538	1,51,490	21,142	70,795
Percentage of reads with non-ATGC bases	0.15%	0.46%	0.07%	0.24%
Total number of bases	3,35,18,30,036	3,35,18,30,036	2,94,07,30,948	2,94,07,30,948
Total number of HQ bases	3,18,35,14,673	3079828686	2,88,86,69,480	2,88,44,62,871
Percentage of HQ bases	94.98%	91.88%	98.23%	98.09%
Total number of non-ATGC bases	54,317	38,94,084	24,353	1,58,213
Percentage of non-ATGC bases	0.00%	0.12%	0.00%	0.01%

**Table 3:** Statistics of SSRs identified in vetiver transcripts.

Total number of sequences examined	73,061
Total size of examined sequences (bp)	5,80,98,639
Total number of identified SSRs	13,527
Number of SSR containing sequences	11,240
Number of sequences containing more than 1 SSR	1,893
Number of SSRs present in compound formation	825
**Distribution to different repeat type classes**	
**Unit size**	**Number of SSRs**
Mono-nucleotide	5,002
Di-nucleotide	1,821
Tri-nucleotide	6,295
Tetra-nucleotide	248
Penta-nucleotide	91
Hexa-nucleotide	70
