# Supplementary material for: De novo assembly and characterization of root transcriptome in two distinct morphotypes of vetiver, Chrysopogon zizaniodes (L.) Roberty
Source: Sci Rep. 2015 Dec 18;5:18630. doi: 10.1038/srep18630 (PMC4683516; doi:10.1038/srep18630)
Supplement: Supplementary Table S2 [file srep18630-s1.doc]

***De novo* assembly and characterization of root transcriptome in two distinct morphotypes of vetiver, *Chrysopogon zizaniodes* (L) Roberty**

Debasis Chakrabarty^a#^, Puneet Singh Chauhan^a#^, Abhishek Singh Chauhan^a^, Yuvraj Indoliya^a^, Umesh Chandra Lavania^b^, Chandra Shekhar Nautiyal^a^*

^a^**CSIR-National Botanical Research Institute, Rana Pratap Marg, Lucknow 226 001, India**

^b^**Department of Botany, Lucknow University, Lucknow 226 001, India**

*For correspondence (e-mail nautiyalnbri@lycos.com)

Dr. Chandra Shekhar Nautiyal

Division of Plant Microbe Interactions

CSIR-National Botanical Research Institute

Rana Pratap Marg, Lucknow - 226 001, India

Phone: +91-522-2205848

Fax: +91-522-2205839

^#^These authors contributed equally to this work

**Table S2.** List of selected genes and primers used for RT-PCR analysis.

| **Locus ID** | **Primers** |
| --- | --- |
| TR29625|c0_g1_i1 | F: 5’- TCTGGCTCCAATCCACATTC -3’  R: 3’- GCACACTTCTTTGCTGTTGC -5’ |
| TR18509|c0_g1_i1 | F: 5’- TTTCAGACACACCAGCAACC -3’  R: 3’- CCAGGAACCCAGTCAAAATG -5’ |
| TR22969|c0_g1_i1 | F: 5’- AATTGTATGGCCTGCTCCAC -3’  R: 3’- CACATACAAGCTGTCCCTAACG -5’ |
| TR23979|c0_g1_i1 | F: 5’- AGCAGGGTGTGTTTGTGAAG -3’  R: 3’- ACTTGCCCCCATTCTCTTTC -5’ |
| TR18928|c0_g1_i1 | F: 5’- TGGTCAGTTCAAAGCAGAAGG -3’  R: 3’- TGCAAGAGGGGACTTGTTTC -5’ |
| TR26232|c0_g1_i1 | F: 5’- TGCATGCTATGGTGGAACAG -3’  R: 3’- ACAAGTCCATAACGCCCATC -5’ |
| TR30426|c0_g2_i1 | F: 5’- CCTTTGACAGCCCATAATCC -3’  R: 3’- CGAGTTCCTGCTCATGTTCC -5’ |
| TR12988|c0_g1_i4 | F: 5’- TGATGGCAAGGAGGGTATTG -3’  R: 3’- TTCAGCGCACTATCCATGTC -5’ |
| TR36389|c0_g1_i1 | F: 5’- CGAGGTTCAGCTTTCCATTC -3’  R: 3’- GCGATGATGAGGATGTTCTG -5’ |
| TR22581|c0_g1_i1 | F: 5’- CAGCAAGGAAAGGAGCAAAC -3’  R: 3’- ATATAAGCGTTGGGCTGGTG -5’ |
| TR15490|c0_g1_i1 | F: 5’- ATACCGAGGTGTCAATGCTG -3’  R: 3’- AGCTTCTTGGTTGAGTGCTG -5’ |
| TR21331|c0_g1_i1 | F: 5’- ATGCCTGGTTCATTGGTCTC -3’  R: 3’- AAGGCTGGTAGCAAAACGAG -5’ |
| TR31737|c1_g1_i1 | F: 5’- TGCCTGAAGACCAACATCTG -3’  R: 3’- GCTGAGAGTTTTCCCTTTGG -5’ |
| TR35777|c3_g1_i2 | F: 5’- CCCAAATATAACCGCTGTGC -3’  R: 3’- ACTTCCACAAGGCATCGTTC -5’ |
| TR4378|c0_g1_i1 | F: 5’- CGCTGCTTCTGCTTGTTG -3’  R: 3’- TGCGCCTCGAAGTAGAGC -5’ |
| TR11879|c0_g1_i1 | F: 5’- TCATAGGCTTATGGGTTGAGG-3’  R: 3’- AGCTGGATTGCAAGAGGTTC-5’ |
| TR33547|c0_g1_i1 | F: 5’- GGTGGTAGGAGAAAGCATCG-3’  R: 3’- TCTCCACCACAATGATGTCG-5’ |
| TR42436|c0_g1_i1 | F: 5’- TGAAGTCGTAGCAGGTGTCG-3’  R: 3’- GCCCATGCTGTACCTCGT-5’ |
| TR48901|c0_g1_i1 | F: 5’- CCACCGGAGAGGAAGTACAG-3’  R: 3’- GCAGAGCAACCACCCAATAG-5’ |
| TR34338|c1_g1_i3 (Actin) | F: 5’- GCCTTCCTTCATTGGTATGG-3’  R: 3’- ACCAGGGAACATTGTTGAGC-5’ |
